# Functional and Radiological Outcomes of Short-Segment Fixation With Intermediate Screws for Thoracolumbar Spine Fractures

**DOI:** 10.7759/cureus.75653

**Published:** 2024-12-13

**Authors:** Vaibhav Jain, Vishal Champawat, Manish Rajpoot, Abhishek Sarkar, Rahul Verma, Vijendra Parmar, Anagh Saha

**Affiliations:** 1 Department of Orthopaedics, Gandhi Medical College, Bhopal, Bhopal, IND

**Keywords:** burst fractures, intermediate screws, short-segment fixation, spinal stabilization, spinal trauma, thoracolumbar fractures, vertebral body fractures

## Abstract

Introduction

Thoracolumbar fractures, particularly burst fractures, represent a significant health concern due to their prevalence and functional impact. This study evaluates the efficacy of short-segment posterior fixation with intermediate screw instrumentation in treating unstable thoracolumbar fractures.

Methods

A prospective study was conducted from July 2022 to December 2023, including 26 patients with traumatic thoracolumbar fractures. Surgical intervention was indicated for patients with a thoracolumbar injury classification and severity (TLICS) score >4. Functional outcomes were assessed using the visual analog scale (VAS), Oswestry Disability Index (ODI), and the American Spinal Injury Association (ASIA) grading system. Radiological outcomes, including the local kyphotic angle (LKA) and anterior vertebral body height ratio (AVBHR), were evaluated preoperatively, postoperatively, and at follow-ups.

Results

Significant improvements in functional and radiological parameters were observed over 24 weeks. The mean VAS score reduced from 7.50 ± 0.58 preoperatively to 1.42 ± 0.50 (p < 0.001), and the mean ODI improved from 42.23 ± 3.54 to 16.12 ± 3.09 (p < 0.001). Neurological improvements were seen in ASIA grades B-D, with no new deficits. Radiologically, the mean LKA improved from 19.73 ± 1.59° preoperatively to 8.46 ± 1.33° at 24 weeks (p < 0.001), and the AVBHR increased from 34.08 ± 2.25% to 86.64 ± 0.83% (p < 0.001). No implant failures were reported.

Conclusion

Short-segment fixation with intermediate screws provides effective stabilization and significant functional and radiological improvements in thoracolumbar fractures. It minimizes intraoperative morbidity and preserves motion segments, making it a viable alternative to long-segment fixation, particularly for non-complex fractures. Further randomized controlled trials are recommended to validate these findings.

## Introduction

Spinal fractures, particularly thoracolumbar vertebral body fractures, are a significant global health concern, with an estimated annual incidence of 15-40 cases per million globally and approximately 21 cases per million in South Asia [1,2]. In India, around 20,000 new spinal cord injury cases are reported annually, accounting for about 6% of all fractures. Thoracolumbar fractures constitute 21% to 58% of all spinal fractures, with the dorso-lumbar junction being particularly vulnerable due to its high mobility and biomechanical stress. This region, located between the rigid thoracic spine (stabilized by the rib cage) and the more mobile lumbar spine, is prone to injury in traumatic events [3]. Neurological injury occurs in approximately 15-20% of these patients [4].

A common type of thoracolumbar fracture is the burst fracture, resulting from axial forces that compromise the anterior and middle vertebral columns. Unstable burst fractures require surgical stabilization, characterized by a reduction in anterior vertebral height of >50%, an increase in kyphotic angle >20 degrees, or more than 50% canal compromise. Neurological progression is a key indicator for surgery [5].

Surgeons prefer the posterior approach due to its ability to provide superior correction and stability after surgery, despite the potential risks of increased blood loss and longer surgical duration [6]. The four-screw short-segment posterior instrumentation has become popular; however, its limitations include potential early implant failure and loss of correction [7,8]. Alternative methods, such as short-segment fixation with pedicle screw augmentation, offer promising outcomes with reduced operative time and blood loss [9,10]. This study aims to assess the functional and radiological outcomes of thoracolumbar unstable fractures treated by short-segment fixation with intermediate screw instrumentation.

## Materials and methods

This prospective study, conducted from July 2022 to December 2023 at a tertiary care hospital, included 26 patients who were diagnosed with traumatic thoracolumbar fractures. Institutional ethical clearance was obtained before the study. Inclusion criteria comprised adult patients (≥18 years) with traumatic thoracolumbar fractures confirmed by plain radiographs. Exclusion criteria included patients who were unwilling to provide consent, those with severe lower limb trauma unfit for orthopedic intervention, and patients with pathological fractures. Informed written consent was obtained from all eligible patients.

Initial patient assessment included a detailed history of the injury, mode of injury, and a thorough clinical and neurological examination. The stability of the spine was assessed, and priority was given to resuscitation and treatment of life-threatening injuries before addressing spinal injuries. Associated skeletal injuries were also examined. A complete neurological examination, including sensation, motor function, and anal tone, was performed and documented. Protection of the spinal column was prioritized. Imaging studies, including anteroposterior and lateral X-rays, computed tomography (CT )scans, and magnetic resonance imaging (MRI), were conducted to assess the extent and nature of the injury. Fractures were classified, and the thoracolumbar injury classification and severity score (TLICS) score was calculated. Surgical intervention was indicated for a TLICS score >4. American Spinal Injury Association (ASIA) grading was performed, and treatment options were discussed with patients and their families. All patients underwent short-segment posterior stabilization with index vertebra fixation.

Surgical technique

After general anesthesia was administered, patients were positioned prone with bolsters under the chest and iliac crests. The surgical procedure began with routine disinfection and draping of the surgical site. Intraoperative fluoroscopy was used to locate the fracture. A midline posterior incision was made, and the erector spinae muscles were carefully stripped bilaterally. The fascia, supraspinal ligament, and surrounding soft tissues were removed to expose the spinous process and lamina of the affected vertebra. The bilateral lamina and facet joints of the fractured vertebra and its adjacent upper and lower levels were then exposed.

The pedicles were identified by the intersection of the transverse process and superior facet. A rongeur was used to remove cortical bone, and K-wires were inserted into the pedicles, with their position confirmed under fluoroscopic guidance. The pedicles were probed to ensure no cortical violation, followed by tapping and insertion of appropriately sized titanium pedicle screws (5.5-6.5 mm in diameter, 40-45 mm in length).

For short-segment fixation, screws were placed in the vertebrae above and below the fractured level, and intermediate screws were inserted in the fractured vertebra, avoiding pedicles with fractures. Kyphosis reduction was achieved through rod over-contouring or distraction. Decompression via laminectomy was performed in cases with neurological deficits or spinal canal compromise.

Postoperatively, patients received intravenous antibiotics for five days, followed by oral antibiotics. Physiotherapy commenced on the first day, with mobility encouraged starting on day 1 using a Knight-Taylor brace. Suture removal was done on the fourteenth day. Patients were closely monitored in outpatient follow-ups.

Postoperative assessment

Follow-up evaluations were conducted in the outpatient department at the sixth, twelfth, and twenty-fourth weeks.

Functional assessment

At each follow-up, functional outcomes were assessed using the visual analog scale (VAS), Oswestry Disability Index (ODI), and the ASIA impairment scale grade.

Radiological assessment

Radiological parameters, including the local kyphotic angle (LKA) and anterior vertebral body height ratio (AVBHR), were measured preoperatively, postoperatively, and during follow-up.

Local Kyphotic Angle (LKA)

LKA was determined by measuring the angle between a line parallel to the superior endplate of the vertebra above the fracture and a line parallel to the inferior endplate of the vertebra below the fracture. This measurement was performed on standard lateral radiographs using a protractor. A line was drawn through the superior endplate of the cranial vertebra and the inferior endplate of the caudal vertebra. A perpendicular line was drawn from both of them and the angle was measured between their intersection.

Anterior Vertebral Body Height Ratio (AVBHR)

The AVBHR was calculated by dividing the anterior height of the injured vertebral body by the average anterior height of the adjacent upper and lower vertebral bodies. For the AVBHR, the anterior vertebral height was measured and the result was obtained by putting the values in the following equation

\[ AVHR = \left[ \frac{a}{(b + c) / 2} \right] 100 \]

where “a” is the index vertebra, “b” is the upper vertebra, and “c” is the lower vertebra 

Statistical analysis

All data were entered into Microsoft Excel 2010 (Microsoft Corporation, Redmond, WA, US). Descriptive statistics were expressed as mean ± standard deviation (SD). Frequency distribution and percentages were used to analyze gender and age distribution. The chi-square test or Fisher's exact test was applied to assess associations. Time intervals for various parameters were compared using repeated measures analysis of variance (ANOVA) while non-parametric data, such as ASIA grades, were analyzed using the Friedman test. For all the tests, a p-value <0.05 was considered statistically significant. Statistical analyses were conducted using SPSS v 19 (IBM Corp., Armonk, NY, US).

## Results

Among the 26 patients included in the study, 69.2% (18) were male and 30.8% (8) were female. Most cases (26.9%; 7) were within the 21-30-year age group, followed by 19.2% (5) in the 31-40-year age group, indicating that young adults were the most affected (Table 1 ). The most common mechanism of injury was a fall from height, accounting for 61.54% (16) of cases, which reflects the high incidence of traumatic spinal injuries in this population.

**Table 1:** Distribution of demographic and general characteristics of patients enrolled in the study (n = 26)

CHARACTERISTICS	NUMBER (n)	PERCENTAGE (%)
AGE GROUP (IN YEARS)		
11-20 Years	4	15.4
21-30 Years	7	26.9
31-40 Years	5	19.2
41-50 Years	3	11.5
51-60 Years	5	19.2
61-70 Years	1	3.8
71-80 Years	1	3.8
Total	26	100.0
GENDER		
Male	18	69.2
Female	8	30.8
Total	26	100.0
MECHANISM OF INJURY		
Fall from height	16	61.54
Road traffic accident	10	38.46
Total	26	100.0
FRACTURE LEVEL		
D11	3	11.54
D12	7	26.92
L1	9	34.62
L2	3	11.54
L3	2	7.69
L4	2	7.69
Total	26	100.0
TLICS (Thoracolumbar Injury Classification and Severity)		
5	6	23.08
6	6	23.08
7	11	42.31
8	2	7.69
9	1	3.85
TOTAL	26	100.0
ASSOCIATED INJURIES		
Calcaneus fracture	04	15.38
American Spinal Injury Association (ASIA) Impairment Scale		
A	8	30.77
B	3	11.54
C	3	11.54
D	5	19.23
E	7	26.92
Total	26	100
FRACTURE TYPE		
Burst	12	46.15
Wedge	14	53.85
Total	26	100

**Figure 1:**
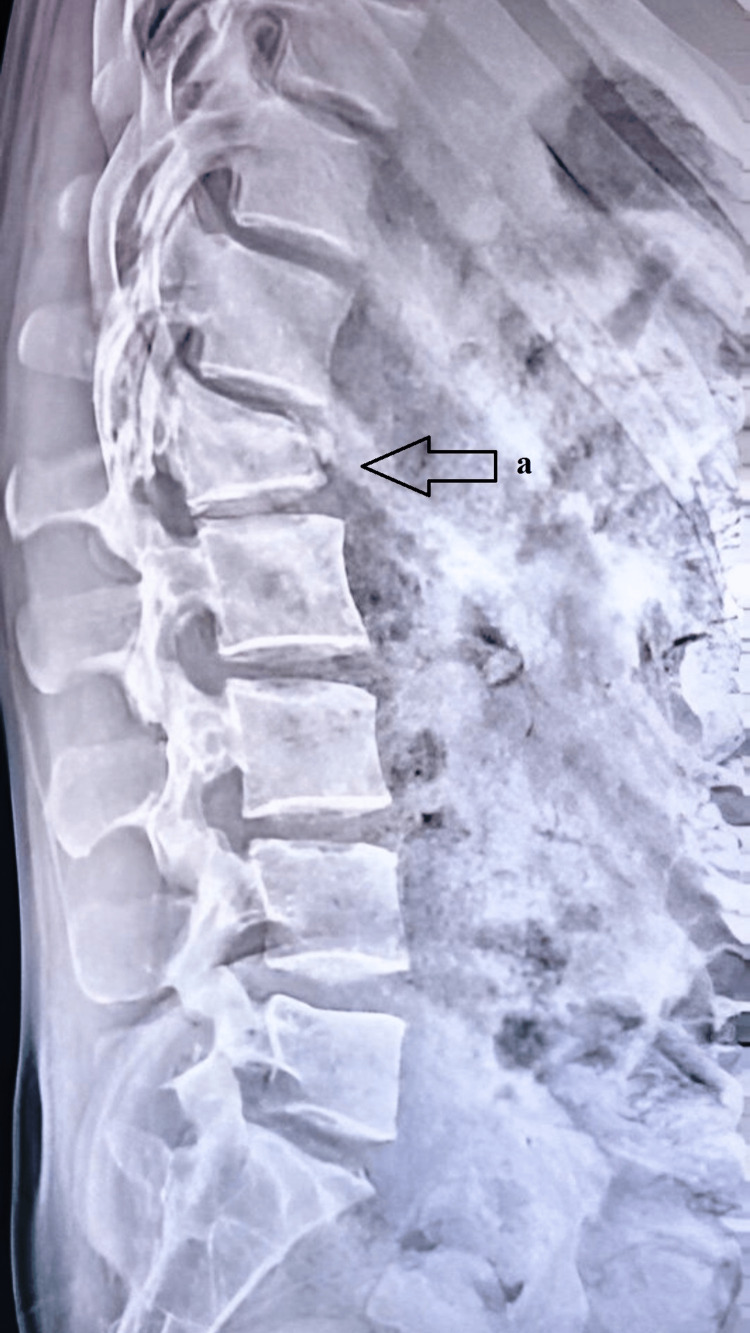
Radiograph showing a fracture in the L1 vertebral body a: denoting an L1 vertebral body fracture on the radiograph

**Figure 2:**
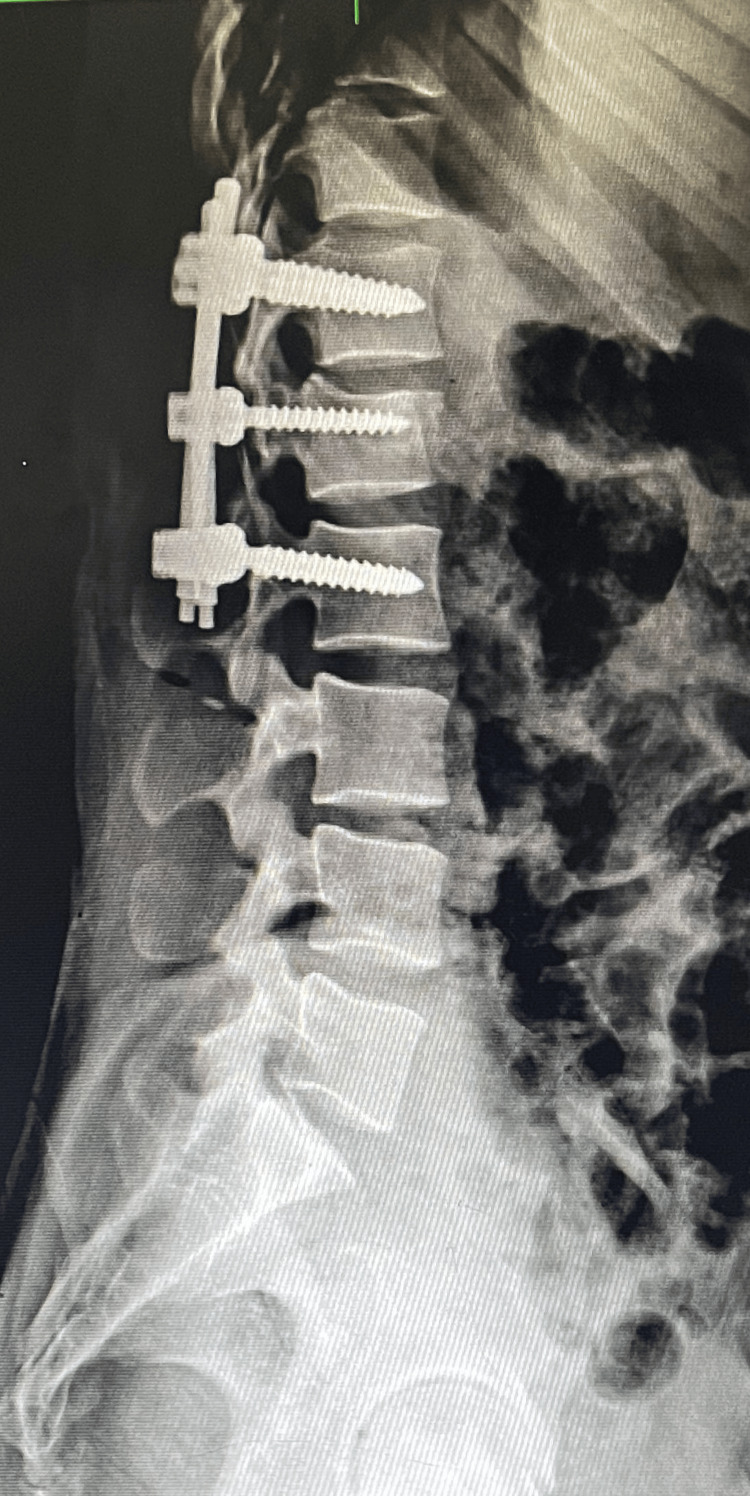
Postoperative radiograph showing short-segment fixation with an intermediate screw in the fractured L1 vertebra

**Figure 3:**
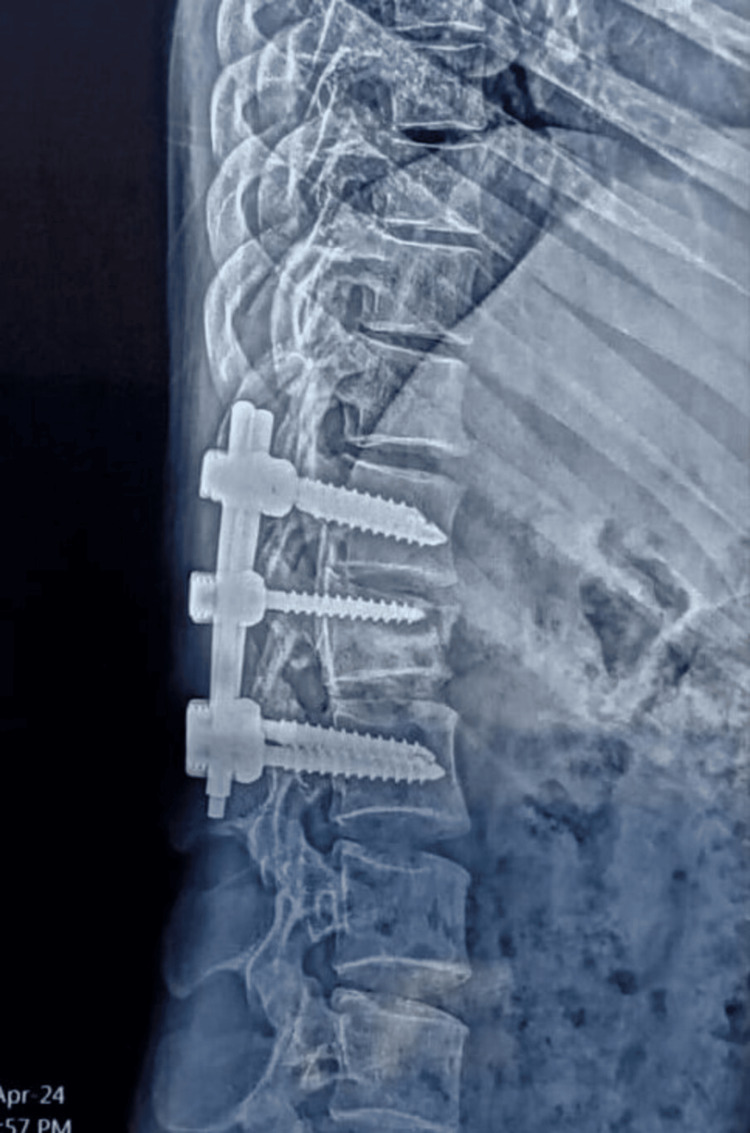
The 24-week follow-up radiograph of the L1 fracture fixed with short-segment fixation with an intermediate screw

The most frequently involved vertebral levels were L1 (34.62%; 9) and D12 (26.92%; 7), consistent with the thoracolumbar junction being a biomechanically vulnerable region (Figures 1-3). Wedge compression fractures were the most prevalent fracture pattern, observed in 53.85% (14) of cases, and the most common TLICS was 42.31% (7), indicating the necessity for surgical intervention in these cases. Additionally, the most frequently associated injury was a calcaneal fracture, indicative of high-energy trauma.

The average blood loss during surgery was 305.96 ± 22.716 mL, reflecting the procedure's relatively controlled intraoperative parameters.

Clinical outcomes

Visual Analog Scale (VAS)

The mean VAS score for patients undergoing short-segment fixation was 7.50 ± 0.583 preoperatively, indicating severe pain levels (Table 2). Postoperatively, there was a significant and progressive reduction in pain at each follow-up. The mean VAS score decreased to 3.96 ± 0.871 (n=26) at 6 weeks, further reduced to 1.62 ± 0.637 (n=26) at 12 weeks, and reached 1.42 ± 0.504 (n=26) at 24 weeks. This reduction in VAS scores from the preoperative period to the final follow-up at 24 weeks was statistically significant, reflecting a consistent and substantial decrease in pain over time. These results highlight the effectiveness of short-segment fixation in providing sustained pain relief, significantly improving patient comfort and quality of life postoperatively.

**Table 2:** . Descriptive statistical analysis of functional and radiological parameters (n =26) VAS: visual analogue scale; ODI: Oswestry Disability Index; LKA: local kyphotic angle; AVBHR: anterior vertebral body height ratio; ° : Degrees; %: percentage

(n=26)	PREOPERATIVE VALUES		IMMEDIATE POSTOPERATIVE VALUES		FINAL FOLLOW-UP VALUES		REPEATED MEASURES ANOVA (p-value)
	Mean	SD	Mean	SD	Mean	SD	
VAS	7.50	.583	3.96	.871	1.42	.504	<0.001
ODI	42.23	3.547	37.23	2.405	16.12	3.090	<0.001
LKA (°)	19.73	1.589	5.96	1.399	8.46	1.334	<0.001
AVHR (%)	34.080769	2.2466009	88.142	.5658	86.635	.8381	<0.001

Neurological Outcomes (ASIA Grade)

Preoperatively, the neurological status of the patients was classified using the ASIA grading system, with 30.77% (9) patients in grade A, 11.54% (3) in grade B, 11.54% (3) in grade C, 19.23 % (5) in grade D, and 26.92 % (7 )in grade E. The high prevalence of ASIA grade A highlights the severity of neurological deficits in a significant proportion of patients. Postoperatively, neurological improvement was observed in some cases. Among the grade B patients, 1 (3.84%) showed improvement to grade C. Of the grade C patients, 7.69% (2) improved, 3.84% (1) progressed to grade D, and another to grade E. Notably, 15.38% (4) patients in grade D demonstrated significant recovery, achieving grade E status. However, no improvement was observed in patients initially classified as grade A (30.77%, 9); all (26.92%; 7) patients in grade E maintained their preoperative status. These findings indicate that while neurological improvement was achievable in patients with some preserved function (grades B to D), those with complete spinal cord injury (grade A) showed no recovery, underscoring the prognostic implications of the initial ASIA grade.

Oswestry Disability Index (ODI)

The mean ODI score was 42.23 ± 3.547 % (n=26) preoperatively, indicating that most patients fell into the category of severe disability. Postoperatively, there was a statistically significant improvement at each follow-up interval. At 6 weeks, the mean ODI score decreased to 37.23 ± 2.405 % (n=26), further improving to 31.42 ± 2.176 % (n=26) at 12 weeks, and reaching 16.12 ± 3.090% at 24 weeks. This progressive reduction in ODI scores reflects a substantial improvement in patient disability levels over time, with the majority transitioning from severe disability to minimal disability by the 24-week mark. These results highlight the effectiveness of the intervention in enhancing patients' functional status and quality of life.

Local Kyphotic Angle (LKA)

**Figure 4:**
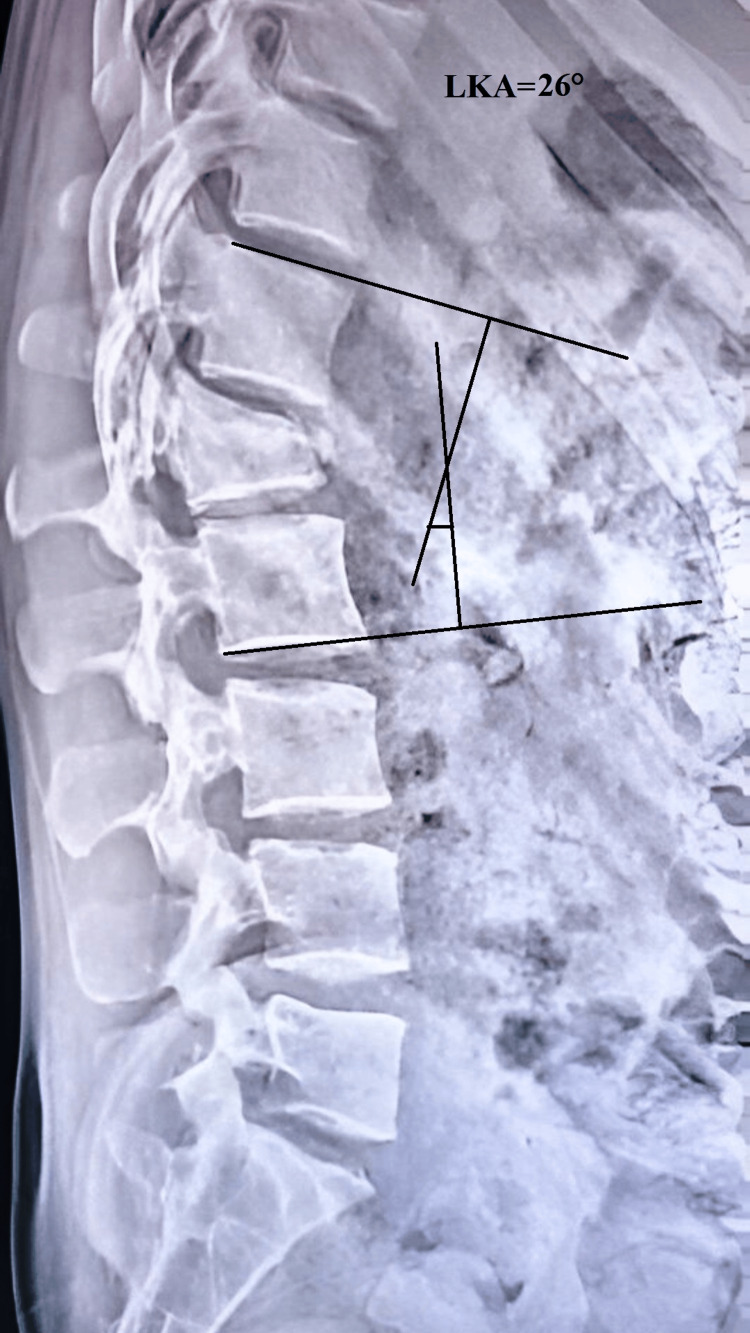
Radiograph showing measurement of the local kyphosis angle Radiograph showing measurement of the preoperative LKA of 26 degrees at the L1 fracture. The local kyphosis angle was determined by measuring the angle between a line parallel to the superior endplate of the vertebra above the fracture and a line parallel to the inferior endplate of the vertebra below the fracture. LKA: local kyphosis angle; ° : degrees

**Figure 5:**
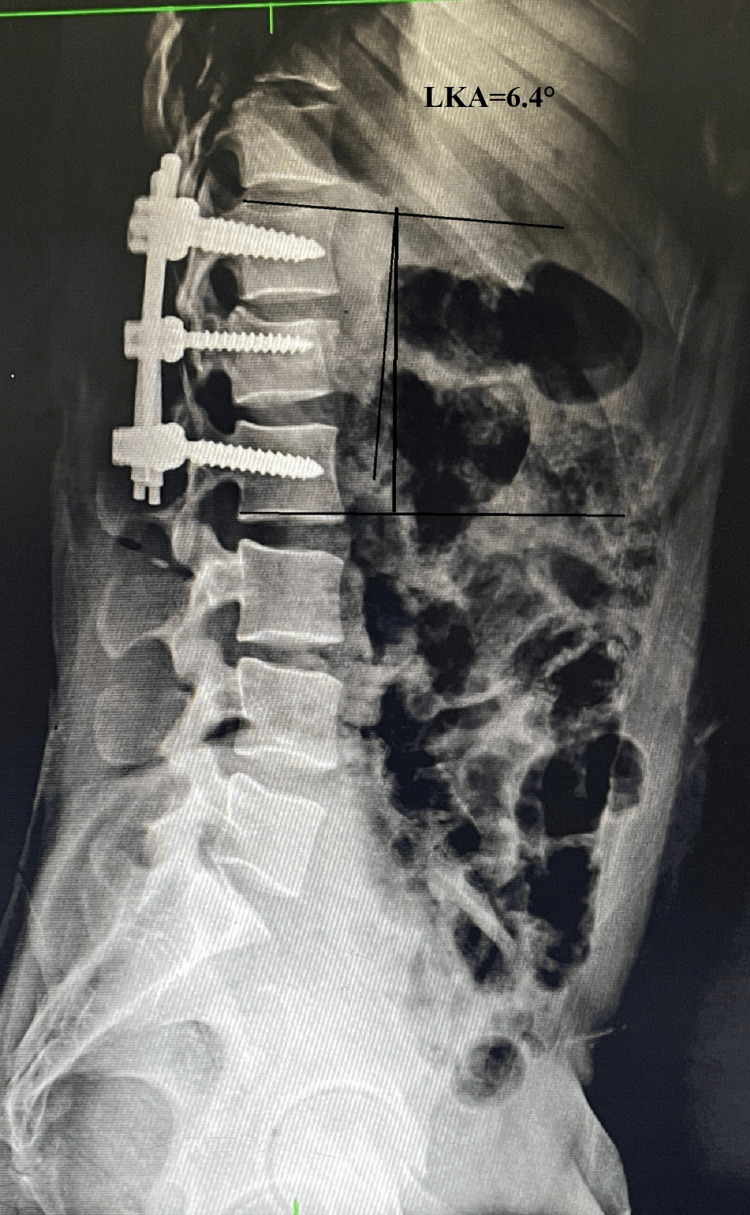
Radiograph showing post-operative measurement of the LKA Radiograph showing postoperative improvement in kyphosis after short-segment fixation with an intermediate screw at L1. The LKA was 6.4 degrees. LKA: local kyphosis angle; ° : degrees

**Figure 6:**
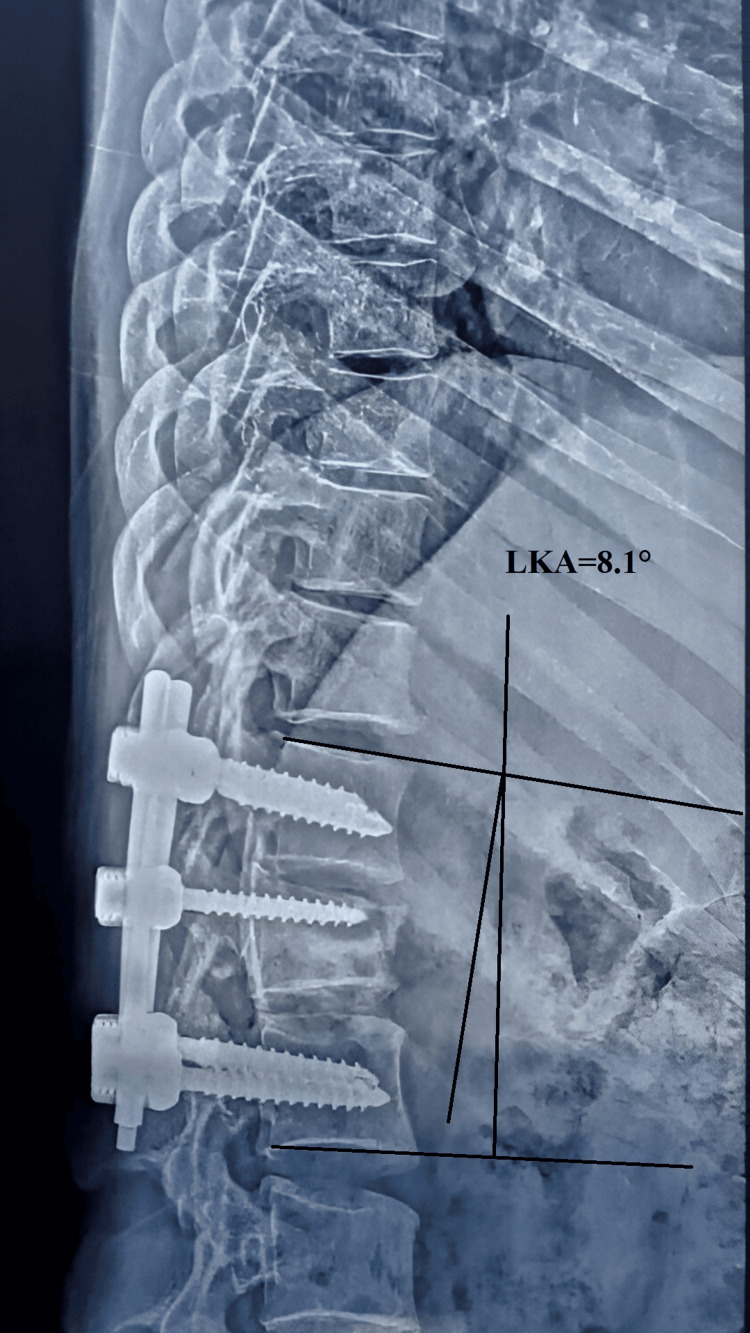
The 24-week follow-up radiograph showing measurement of the LKA Radiograph showing maintenance of reduction with marginal change in the LKA at the 24-week follow-up. The local LKA was 8.1 degrees. LKA: local kyphosis angle; ° : degrees

The mean LKA was 19.73° ± 1.589° (n=26) preoperatively, indicating a significant degree of kyphotic deformity. Following surgery, the LKA improved markedly to 5.96° ± 1.399° (n=26), demonstrating successful correction of the deformity. This correction was largely maintained over time, with mean LKA values of 6.42° ± 1.528° (n=26) at 6 weeks, 7.62° ± 1.329° (n=26) at 12 weeks, and 8.46° ± 1.334° ( n=26) at 24 weeks (Figures 4-6). The reduction in LKA from the preoperative period to the final follow-up at 24 weeks was statistically significant. These findings highlight that the surgical intervention achieved a substantial correction of kyphosis, which was effectively maintained over the follow-up period with minimal loss of correction, underscoring the durability and stability of the procedure.

Anterior Vertebral Body Height Ratio (AVBHR)

**Figure 7:**
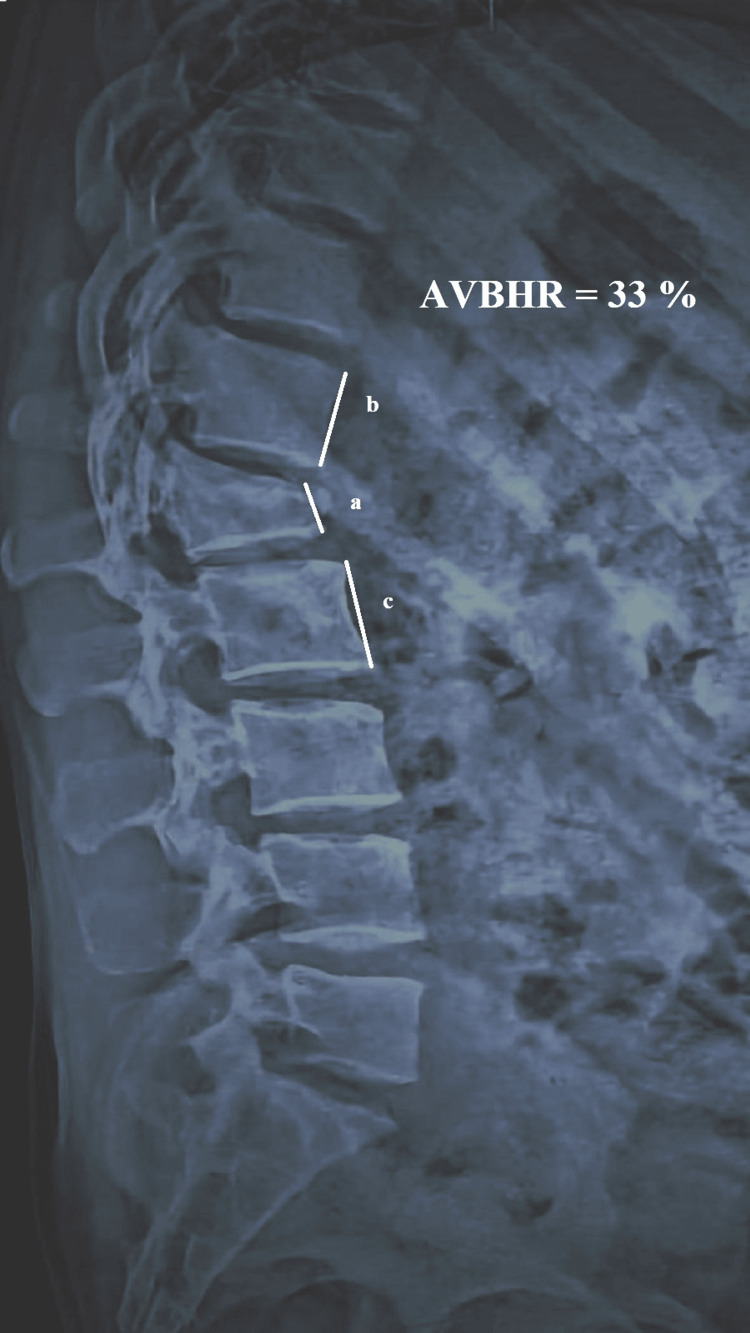
Radiograph showing preoperative measurement of AVBHR Radiograph showing the measurement of preoperative AVBHR as 33%. AVBHR is calculated by dividing the anterior height of the injured vertebral body by the average anterior height of the adjacent upper and lower vertebral bodies. AVBHR: anterior vertebral body height ratio; a: anterior margin height of the injured vertebral body; b: anterior margin height of the adjacent upper vertebral body; c: anterior margin height of the adjacent lower vertebral body; %: percentage

**Figure 8:**
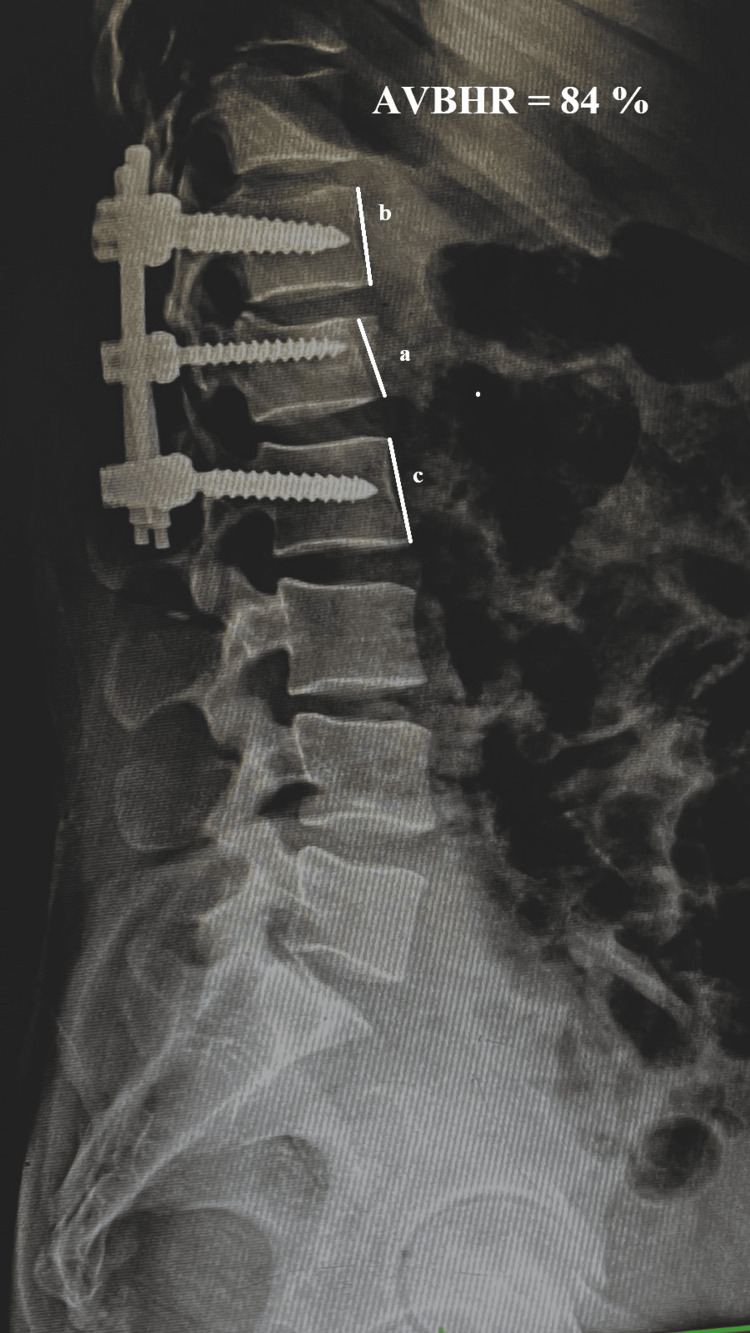
Postoperative radiograph with measurement of the anterior vertebral body height ratio Radiograph showing postoperative improvement in the anterior vertebral height with an AVBHR of 84%. AVBHR: anterior vertebral body height ratio; a: anterior margin height of the injured vertebral body; b: anterior margin height of the adjacent upper vertebral body; c: anterior margin height of the adjacent lower vertebral body; %: percentage

**Figure 9:**
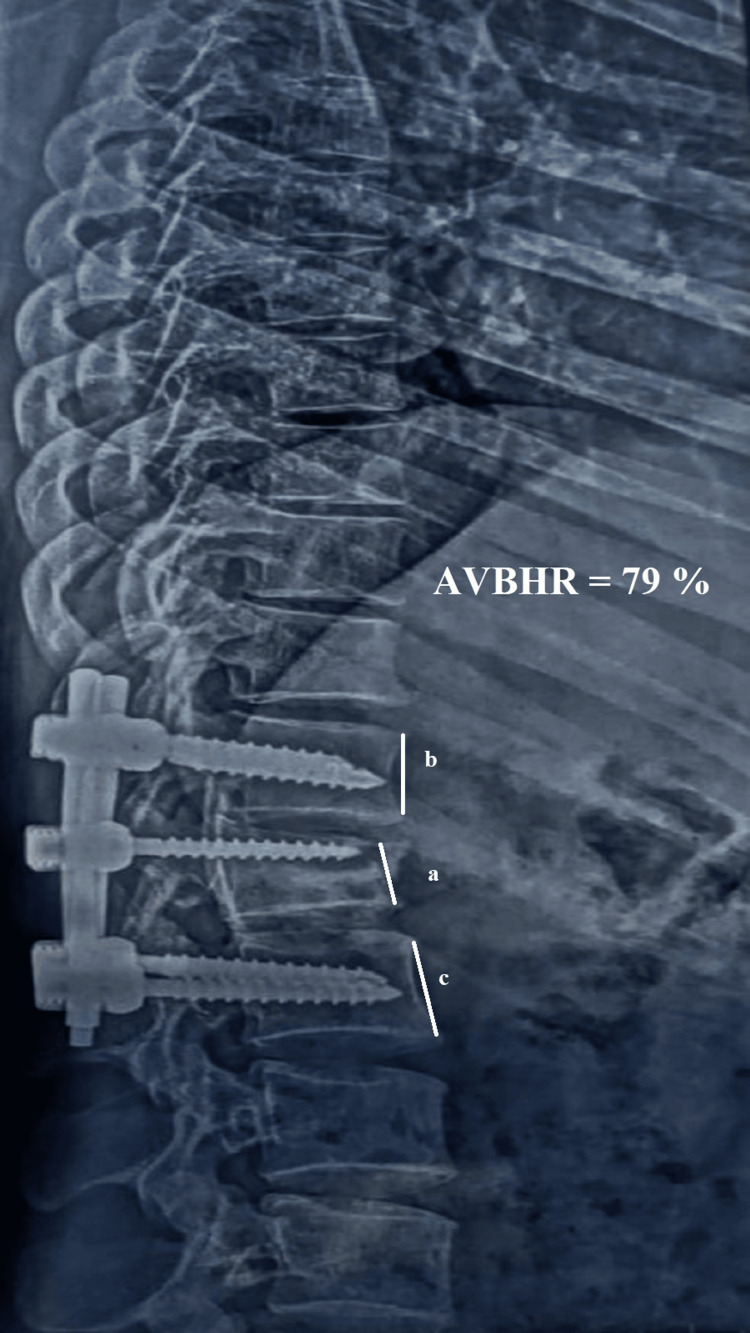
The 24-week follow-up radiograph with measurement of the anterior vertebral body height ratio Radiograph showing maintenance of the vertebra with a slight decrease in height at the 24-week follow-up. The AVBHR was 79%. AVBHR: anterior vertebral body height ratio; a: anterior margin height of the injured vertebral body; b: anterior margin height of the adjacent upper vertebral body; c: anterior margin height of the adjacent lower vertebral body; %: percentage

The mean AVBHR showed a significant improvement from 34.08% ± 2.25% (n=26) preoperatively to 88.14% ± 0.658% (n=26) immediately after surgery, indicating substantial restoration of vertebral height (Figures 7-9). Over the follow-up period, there was a slight and gradual reduction in AVBHR, with values of 87.88% ± 0.680% (n=26) at 6 weeks, 87.20% ± 0.813% (n=26) at 12 weeks, and 86.64% ± 0.838% (n=26) at 24 weeks. Despite this minor decrease, the improvement in AVBHR from preoperative levels remained statistically significant throughout, reflecting successful and durable restoration of vertebral height postoperatively, which was well-maintained until the final follow-up. These findings emphasize the procedure's efficacy in correcting vertebral height and sustaining structural integrity over time.

Overall, the findings demonstrate a statistically significant improvement in clinical, radiological, and functional outcomes over 24 weeks following short-segment fixation.

## Discussion

In this study, no cases of implant failure were reported, reflecting the effectiveness of short-segment constructs with intermediate screws in preventing hardware-related complications. Kose et al. and Jindal et al. highlighted the importance of fracture-level screws in reducing correction loss and implant failure rates, particularly in highly unstable fractures [11,12]. The findings from this study reinforce these conclusions, demonstrating the long-term stability and efficacy of short-segment constructs.

The thoracolumbar spine is highly susceptible to injury due to its unique anatomical and biomechanical properties. Located at the transitional junction between the less mobile thoracic spine and the more flexible lumbar spine, this region is particularly vulnerable to high-energy trauma such as falls and motor vehicle accidents [4]. These injuries often occur in younger individuals, leading to significant functional and socioeconomic consequences. Treatment of thoracolumbar fractures aims to achieve a pain-free, stable spine and restore maximum function.

This study reviewed 26 patients with thoracolumbar fractures, with the highest incidence observed in the 21-30 age group (26.9%; 7) and a mean age of 38.12 ± 16.8 years, consistent with previous studies, such as Biakto et al., who reported a mean age of 35 ± 13 years [13]. Males accounted for 69.2% of the cases, and the most common mechanism of injury was falls from height. These findings align with established trends, indicating a higher prevalence of thoracolumbar fractures among active young males engaged in occupations or recreational activities that predispose them to trauma.

The L1 vertebral level was most frequently affected, observed in 34.62% (9) of cases. This predilection is attributable to the transitional location of L1, where thoracic kyphosis shifts to lumbar lordosis, making it susceptible to axial and shear forces during trauma. These findings are consistent with research by Mustard et al. [14]. Wedge compression fractures were the most common fracture pattern, identified in 53.85% (14) of cases, with a thoracolumbar injury severity score of 7 observed in 42.31% (11) of cases. Regarding neurological impairment, 30.77% (8) of patients were classified as ASIA grade A, followed by 26.92% (7) as ASIA grade E. The most frequently associated injury was a calcaneal fracture, consistent with earlier studies by Worsham et al. [15], which reported a 50% prevalence of associated injuries in thoracolumbar trauma.

The mean blood loss during surgery was 305.96 ± 22.7 ml, which aligns with findings by Tezeren et al., who reported mean blood loss of 411 ± 111 ml for short-segment fixation and 550 ± 145 ml for long-segment fixation. Similarly, Nagaty et al. observed reduced operative time and blood loss with short-segment fixation, highlighting its efficiency in minimizing surgical morbidity [16].

Significant improvements in pain and disability outcomes were observed over the follow-up period. The mean VAS score decreased from 7.50 ± 0.58 preoperatively to 1.42 ± 0.50 at 24 weeks postoperatively (p < 0.001), consistent with findings by Necdet et al. and Tarek et al., who reported comparable pain relief between short- and long-segment fixation groups [17,18]. Functional recovery, as assessed by the ODI, showed significant improvement from a mean of 42.23 ± 3.54 preoperatively to 16.12 ± 3.09 at 24 weeks postoperatively (p < 0.001), corroborating results from Zacarias et al., who highlighted the efficacy of short-segment fixation in enhancing functional outcomes [19].

Neurological outcomes, assessed using the ASIA grading system, showed limited improvements in patients with incomplete neurological deficits. One patient improved from grade B to C, one from grade C to D, and one from grade C to E. Additionally, four patients improved from grades D to E. No improvements were observed in patients with ASIA grade A injuries, consistent with findings by Zacarias et al., who reported better outcomes in patients with higher initial ASIA grades [20]. Importantly, no new neurological deficits were observed, underscoring the safety of short-segment fixation techniques.

Radiological outcomes demonstrated significant improvements in the LKA and AVBHR. The mean preoperative LKA of 19.73 ± 1.59° improved to 8.46 ± 1.33° at 24 weeks. Minimal correction loss was observed, with 11.5% of patients experiencing a 1° loss and 15.5% experiencing a 4° loss. These findings align with studies by Gelb et al. and Farrokhi et al., which demonstrated effective kyphotic correction and minimal failure rates with fracture-level screws in short-segment constructs [21,22]. The mean AVBHR improved significantly from 34.08 ± 2.25% preoperatively to 86.64 ± 0.83% at 24 weeks, with results consistent with those of other studies emphasizing the biomechanical advantages of short-segment fixation.

Biomechanically, short-segment posterior fixation with intermediate screws offers enhanced axial and flexion stiffness. Studies by Dick et al. demonstrated a 160% increase in axial stiffness and an 84% increase in flexion stiffness with intermediate screws while Bartanusz et al. reported equivalent stability between intermediate screw constructs and combined anterior-posterior constructs [22,23]. Additionally, Mahar et al. emphasized the protective role of segmental constructs for the anterior column under flexion-extension loads [24].

In terms of implant stability, no cases of implant failure were reported in this study, supporting the efficacy of short-segment constructs with intermediate screws in minimizing hardware-related complications. This finding aligns with research by Kose et al. and Jindal et al., who emphasized the role of fracture-level screws in reducing correction loss and implant failure, particularly in unstable fractures [11,12]. Overall, these findings reinforce the long-term stability, safety, and effectiveness of short-segment fixation with intermediate screws for managing thoracolumbar fractures.

This study is not without limitations. The most common age group in our cohort was 21-30 years, and the results may not be fully generalizable to older individuals, particularly those with associated osteoporosis, which could influence surgical outcomes. Additionally, our study lacked a comparison group without pedicle screw fixation in the fractured vertebra, which could have provided further insights into the specific benefits of this technique.

We compared our findings to previous studies conducted in similar contexts, but our study was not a multicenter investigation. A multicentric approach, with a larger sample size and longer follow-up, would enhance the validity and generalizability of our results. Further research addressing these limitations is essential to strengthen the evidence base and refine clinical recommendations.

## Conclusions

Our study indicates that short-segment fixation with an intermediate screw is as effective as long-segment fixation in managing thoracolumbar fractures, both functionally and radiologically. It achieves adequate correction of the local kyphotic angle (LKA) and restoration of the anterior vertebral body height ratio (AVBHR). Short-segment fixation offers advantages such as reduced intraoperative blood loss, shorter operative times, and preservation of more motion segments. These findings suggest that short-segment fixation with index vertebra instrumentation is a viable alternative, particularly in patients with non-complex fractures.
